# Phosphorylation of Nucleophosmin at Threonine 234/237 is associated with HCC metastasis

**DOI:** 10.18632/oncotarget.5820

**Published:** 2015-10-30

**Authors:** Rachel Hiu Ha Ching, Eunice Yuen Ting Lau, Patrick Ming Tat Ling, Joyce Man Fong Lee, Mark Kin Fai Ma, Bowie Yik Ling Cheng, Regina Cheuk Lam Lo, Irene Oi Lin Ng, Terence Kin Wah Lee

**Affiliations:** ^1^ State Key Laboratory for Liver Research, The University of Hong Kong, Hong Kong, China; ^2^ Department of Pathology, Li Ka Shing Faculty of Medicine, The University of Hong Kong, Hong Kong, China; ^3^ Australian Prostate Cancer Research Centre-Queensland & Institute of Health and Biomedical Innovation, Queensland University of Technology, Brisbane, Australia

**Keywords:** NPM, HCC, CDK1, metastasis, hepatocellular carcinoma, immunohistochemistry

## Abstract

Hepatocellular carcinoma (HCC) is frequently complicated by the occurrence of intrahepatic and extrahepatic metastases, leading to poor prognosis. To improve the prognosis for HCC patients, there is an urgent need to understand the molecular mechanisms of metastasis in HCC. Since protein Serine/Threonine phosphorylation emerges to be an important posttranslational modification critical in signaling process associated with cell proliferation, survival and metastasis, we employed a pair of primary tumor-derived and corresponding lung-metastatic counterparts (PLC/PRF/5-PT and PLC/PRF/5-LM) and aimed to identify these changes using CelluSpot™ Serine/Threonine kinase peptide array. Upon analysis, we found phosphorylated level of nucleophosmin (NPM) at Threonine 234/237 (p-NPM-Thr^234/237^) had remarkably high level in metastatic HCC cells (PLC-LM) than the corresponding primary HCC cell line (PLC-PT). Similar observation was observed in another match primary and their metastatic counterparts (MHCC-97L and MHCC-97H). By immunohistochemical staining, p-NPM-Thr^234/237^ was consistently found to be preferentially expressed in metastatic HCCs when compared with primary HCC in 28 HCC cases (*p* < 0.0001). By overexpressing Flag-tagged NPM and its phosphorylation site mutant (Thr234/237A) into low p-NPM-Thr^234/237^ expressing cells (Hep3B and Huh7) using a lentiviral based approach, we demonstrated that p-NPM-Thr^234/237^ is critical in invasion and migration of HCC cells, and this effect was mediated by cyclin-dependent kinase 1 (CDK1). Wild-type NPM was found to physically interact with a metastatic gene, ROCK2, and defective in Thr234/237 phosphorylation decreased its binding affinity, resulting in decrease in ROCK2 mediated signaling pathway. Identification of CDK1/p-NPM/ROCK2 signaling pathway provides a novel target for molecular therapy against HCC metastasis.

## INTRODUCTION

Hepatocellular carcinoma (HCC) is the fifth most common malignancy worldwide and is the second most fatal cancer in Southeast Asia and Hong Kong [1]. HCC is frequently complicated by occurrence of intrahepatic and extrahepatic metastases even after surgical resection, thereby leading to poor prognosis. To improve the prognosis of HCC patients, there is an urgent need to understand the molecular mechanism of metastasis in HCC. Metastasis is a complicated process involving activation of multiple kinase cascades. Despite the recent advances in biomedical technologies, the molecular mechanism underlying cancer metastasis remains largely unclear.

As a continual pursuit in search of molecular mechanism of HCC metastasis, we employed a pair of primary and its corresponding metastatic lung counterparts (PLC/PRF/5-PT and PLC/PRF/5-LM) by orthotropic injection of parental PLC/PRF/5 cell line into the liver of the SCID mice. These two matched HCC cell lines share the same genetic background but differ in invasive ability which was evidenced by wound healing and cell invasion assays. These two cell lines provide an indispensable tool to study HCC metastasis. Since protein Serine/Threonine phosphorylation emerges to be an important posttranslational modification critical in signaling process associated with cell proliferation, survival and metastasis [2], we aimed to identify its phosphorylated substrate and related pathways which is critical in HCC metastasis. Using CelluSpot™ Serine/Threonine kinase peptide array analysis, we compared the phosphorylation profiling of the two matched HCC cell lines and found phosphorylated level of nucleophosmin (NPM) at Threonine 234/237 (p-NPM-Thr^234/237^) had remarkably high level in metastatic HCC cells (PLC-LM) than the corresponding primary HCC cell line (PLC-PT).

Nucleophosmin (NPM), also known as B23, and located on chromosome 5q35, is a protein of 35–40 KD was originally identified as a non-ribosomal nucleolar phosphoprotein found at high levels in the granular regions of the nucleolus, and shuttles between the nucleus and cytoplasm during the cell cycle [3]. NPM has been implicated in multiple cellular functions including ribosomal protein assembly and transport [3], centrosome duplication [4], and cell cycle progression [5] and programmed cell death [6] by direct or indirect mechanism. Although an elevated level of NPM is often found in certain types of carcinomas including HCC [7]; and has been proposed to be a marker for gastric, colon, ovarian and prostate cancer [3, 6, 8], its oncogenic role is far from fully understood.

Protein phosphorylation and dephosphorylation have been long known to be involved in regulating diverse physiological and pathological processes. NPM exists in cells as a phosphor-protein containing multiple phosphorylation sites [9]. Phosphorylation of NPM at several sites has been previously reported by several different kinases including casein kinase II (CKII), nuclear kinase II, polo-like kinase (Plk) and cdc type kinases (CDK1/cyclin B, CDK2/cyclin E, and CDK2/cyclin A) during mitotic regulation and centrosome duplication [10, 11]. Accumulating evidence has been shown that phosphorylation of NPM has intimately linked to its proliferative effect and played a regulatory role in cell cycle progression. Recent studies demonstrated that deregulated phosphorylation at certain sites (Ser10 and Ser70) of NPM enhanced cell transformation and leukemic blasts in NOD/SCID mice [11]. However, there are no reports showing phosphorylation events on NPM in pathogenesis of solid carcinoma.

In this study, we found that CDK1 mediated p-NPM-Thr^234/237^ is crucial for HCC metastasis through activation of Rho kinase II (ROCK2) signaling pathway. To the best of our knowledge, this is the first study uncovering how NPM phosphorylation (p-NPM-Thr^234/237^) can influence HCC growth and metastasis, and which may be a novel phosphorylated substrate for prognosis and therapeutic intervention for cancer patients.

## RESULTS

### Identification of p-NPM-Thr^234/237^ as critical phosphorylation event in HCC metastasis

**Figure 1:**
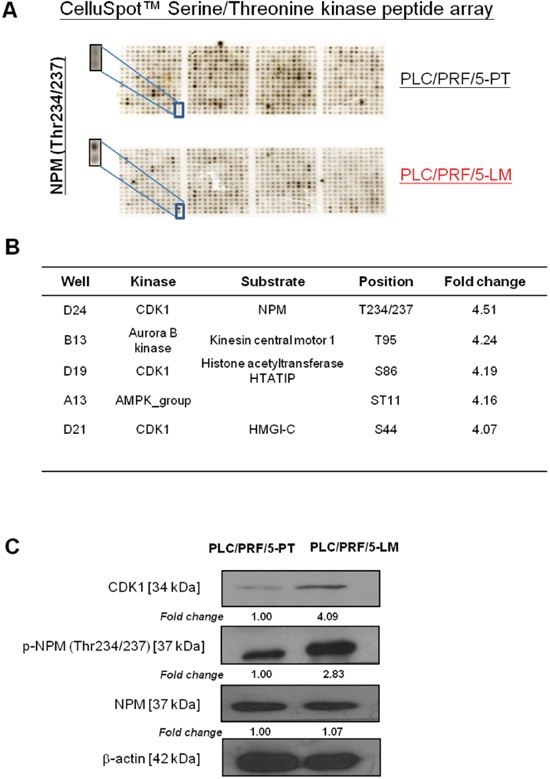
Identification of p-NPM-Thr^234/237^ by CelluSpot™ Serine/Threonine kinase peptide array analysis **A.** p-NPM-Thr^234/237^ was found to be 4.5-fold up-regulation in PLC/PRF/5-LM when compared with PLC/PRF/5-PT. **B.** p-NPM-Thr^234/237^ was found to be phosphorylated by CDK1. **C.** Western blot analysis confirmed up-regulation of p-NPM-Thr^234/237^ and CDK1 in PLC/PRF/5-LM cells.

To understand the molecular mechanism of HCC metastasis, we employed primary and corresponding lung-metastatic counterparts (PLC/PRF/5-PT and PLC/PRF/5-LM), derived by orthotopic injection of parental PLC/PRF/5 cells into the liver of the SCID mice [12]. These two matched HCC cell lines share the same genetic background but differ in their invasive ability. Due to the potential involvement of serine/threonine protein kinase in HCC metastasis, we have adopted a systemic analysis approach to identify corresponding phosphorylated substrate in HCC metastasis by profiling these two matched cell lines using the CelluSpot™ Serine/Threonine kinase peptide array. This glass based array consists of 384 spots in duplicates and each spot consists of 15-mer peptide (substrate) bound to cellulose (Supplementary Figure 1). The exact peptide sequences, corresponding phosphorylation sites and kinases are provided by the manufacturer in a excel sheet. Upon analysis of the phosphorylation profiles by gel documentation system, we identified p-NPM-Thr^234/237^ as the most upregulated phosphorylated substrate in PLC/PRF/5-LM as compared with the PLC/PRF/5-PT cells, with 4.5-fold overexpression (Figure 1A and Supplementary Table 1). NPM phosphorylation at Thr234/237 is the only NPM phosphorylation site that spotted on this phospho-peptide array which is specifically phosphorylated by CDK1 (Figure 1B). Western blot analysis confirmed elevated p-NPM-Thr^234/237^ and CDK1 in PLC/PRF/5-LM cells using a phospho-NPM (Thr234/237) specific antibody (Biolegend) (Figure 1C).

### p-NPM-Thr^234/237^ is associated with HCC metastasis

**Figure 2:**
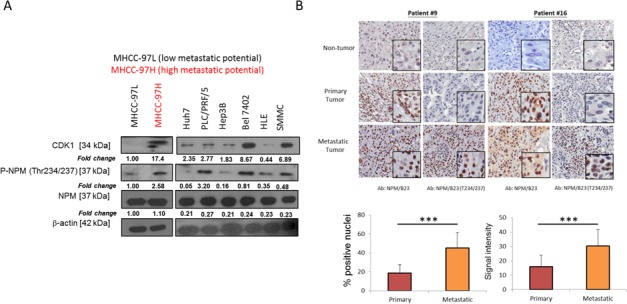
p-NPM-Thr^234/237^ is upregulated in metastatic HCC **A.** CDK1 and its mediated p-NPM-Thr^234/237^ was up-regulated in MHCC-97H when compared with MHCC-97L. In a panel of HCC cell lines, CDK1 and p-NPM-Thr^234/237^ expression was found to be the lowest in Hep3B and Huh7. **B.** In 28 cases of non-tumorous tissues and their matched primary and distinct metastatic tissues, immunohistochemistry revealed a stepwise increase in the expression of p-NPM-Thr^234/237^ from non-tumor, primary HCC to metastatic HCCs and p-NPM-Thr^234/237^ was found to be preferentially expressed in metastatic HCCs when compared with primary HCC in terms of abundance and intensity, respectively (***p < 0.001, student *t* test). There were no difference between primary and metastatic HCCs for NPM expression. Two representative cases (case #9 (adrenal metastasis) and case #16 (spinal metastasis)) were shown.

To eliminate any cell type-specific effect, the role of p-NPM-Thr^234/237^ upregulation in HCC metastasis was further verified in a pair of metastatic HCC cell lines, MHCC-97L and MHCC-97H [13]. Consistently, elevated CDK1 and p-NPM-Thr^234/237^ were also found in the more metastatic HCC cell line, MHCC-97H as compared to the less metastatic cell line, MHCC-97L, while the total NPM level remains unchanged (Figure 2A). In a panel of HCC cell lines including Huh7, PLC/PRF/5, SMMC, Bel 7402, HLE and Hep3B, expression of CDK1 and p-NPM-Thr^234/237^ was variable in which Huh7 and Hep3B were found to have least expression. There was no obvious difference in total NPM expression among these cell lines. To further investigate whether this observation is clinically relevant, we further examined the role of p-NPM-Thr^234/237^ expression in HCC metastasis by examining its expression in 28 cases of non-tumorous tissues and their matched primary and distinct extra-hepatic metastatic tissues. Interestingly, both NPM and p-NPM-Thr^234/237^ exhibited mainly nuclear expression, which were quantitatively evaluated using the Aperio ScanScope CS System. Immunohistochemistry (IHC) revealed a stepwise increase in the expression of NPM-Thr^234/237^ from non-tumor, primary HCC to metastatic HCCs (Figure 2B). p-NPM-Thr^234/237^ was found to be preferentially expressed in metastatic HCCs when compared with primary HCC in terms of abundance and intensity, respectively (*p* < 0.001, student *t* test) (Figure 2B). For NPM, its expression is minimal in non-tumor tissue, but there were no statistical difference between primary and metastatic HCCs.

### p-NPM-Thr^234/237^ is critical in HCC migration and invasion

**Figure 3:**
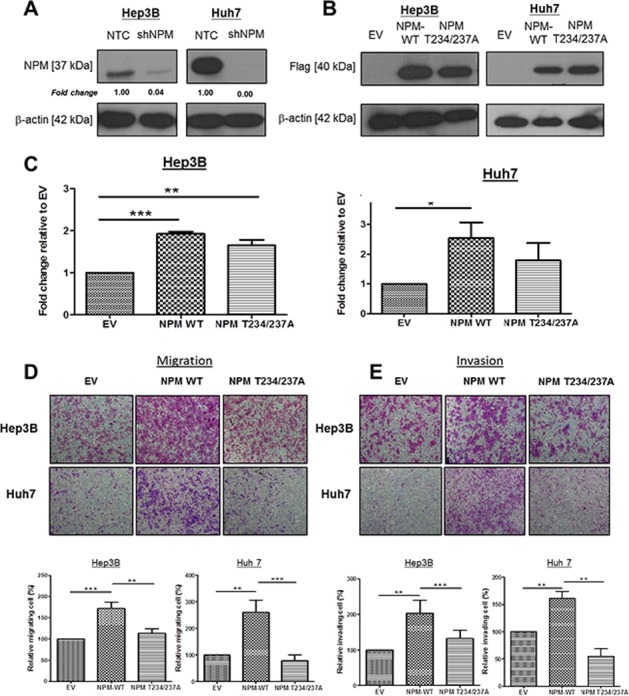
p-NPM-Thr^234/237^ enhanced HCC cell migration and invasion **A.** Endogenous NPM was knocked down by using lentiviral based knockdown approach. By western blot, NPM protein was successfully repressed in Hep3B and Huh7 cells. **B.** NPM-wild type (NPM WT) and NPM Thr234/237A mutant were efficiently overexpressed in Hep3B and Huh7 cells with a lentiviral-based approach, confirmed by protein expression of Flag observed in NPM WT and NPM Thr234/237A transfectants. **C.** BrdU cell proliferation assay demonstrated that NPM wild-type Hep3B and Huh7 cells (NPM WT) had significantly increased cell proliferation rate when compared with control (EV); whereas the proliferative effect is partially abolished in NPM Thr234/237A transfectants (*p < 0.05, **p < 0.01 and ***p < 0.001, student *t* test). **D.** Cell Migration and **E.** invasion assays demonstrated that NPM Thr234/237A mutant transfected Hep3B and Huh7 cells (NPM Thr234/237A) showed reduced migratory and invasion abilities when compared with respective NPM wild-type transfectants (NPM WT) (**p < 0.01 and ***p < 0.001, student *t* test) (magnification × 10).

To further characterize the functional significance of p-NPM-Thr^234/237^ in HCC, we first subcloned Flag-tagged NPM and its phosphorylation site mutant (Thr234/237A) into the pCDH vector (System Biosciences) and overexpressed the gene in low p-NPM-Thr^234/237^ expressing cells (Hep3B and Huh7) using a lentiviral transduction system (System Biosciences). Since Hep3B and Huh7 expressed significant amount of NPM protein, we tried to eliminate the effect of endogenous NPM by knocking down its expression by lentiviral based knockdown approach. By western blot, NPM protein was successfully repressed in Hep3B and Huh7 cells (Figure 3A). Similar levels of the Flag-tagged NPM and phosphorylation site mutant NPM (Thr234/237A) proteins were expressed in Hep3B and Huh7 cells, as determined by western blot analysis (Figure 3B). Stable clones were also established with the pCDH vector alone to serve as a reference for functional comparison. To determine the functional significance of p-NPM-Thr^234/237^ in HCC, we first performed proliferation assay to compare the growth properties of NPM and its phosphorylation site mutant. By BrdU assay, we found significant increase in proliferation in Hep3B and Huh7 cells upon NPM transfection, while its proliferative effect is only partially abolished in Thr234/237A transfectants (Figure 3C). This result showed that p-NPM-Thr^234/237^ is not crucial in regulation of HCC cell proliferation Next, we then tested potential changes in cell migration and invasion *in vitro* upon transfection of NPM and its Thr234/237A mutant. By invasion and migration assays, we found significant increase in cell invasion and migration abilities in NPM transfectants, while this effect is completely abolished in Thr234/237A transfectants. (Figure 3D and 3E). Using *in vitro* scratch assay, we consistently found that NPM WT transfected Hep3B and Huh7 cells had greater migratory ability and this enhancing effect was abolished in Thr234/237 transfectants (Supplementary Figure 2).

### CDK1 phosphorylation of NPM at threonine ^234/237^ is critical in HCC migration and invasion

**Figure 4:**
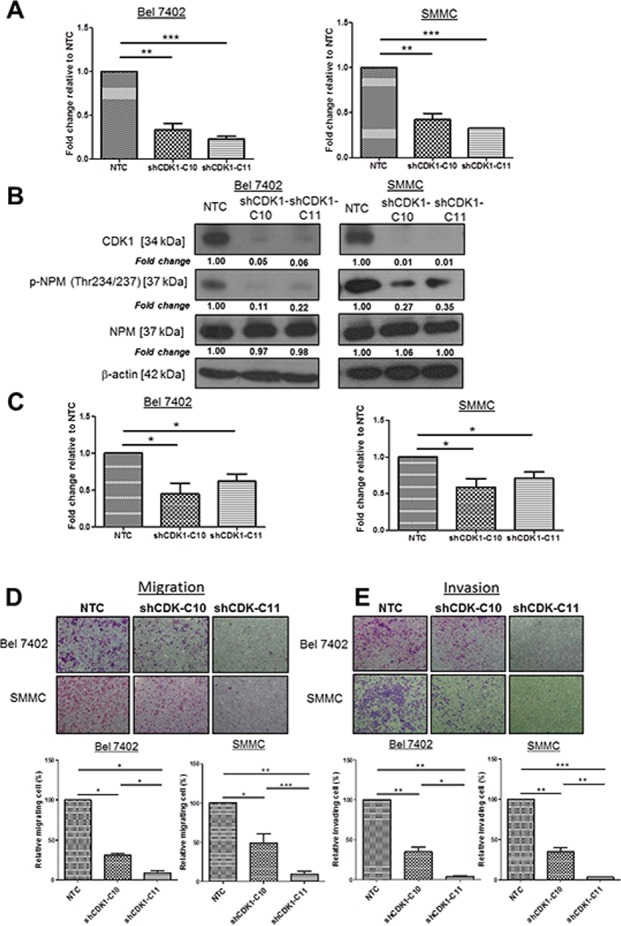
CDK1, upstream kinase of NPM, enhanced HCC cell migration and invasion **A.** CDK1 was efficiently knocked down (C10 and C11) in Bel 7402 and SMMC with a lentiviral-based approach, confirmed by qPCR. **B.** Knockdown of CDK1 reduced NPM phosphorylation but not the total NPM when compared with NTC. **C.** BrdU cell proliferation assay demonstrated that Knockdown of CDK1 in Bel 7402 and SMMC cells (shCDK1-C10 and shCDK1-C11) significantly reduced cell proliferation rate with compared with control (NTC) (*p < 0.05, student *t* test). **D.** Cell migration and **E.** invasion assays demonstrated the number of migrated and invaded cells was significantly reduced in shCDK1-transfected Bel 7402 and SMMC cells (shCDK1-C10 and shCDK-C11) when compared with control (NTC) (*p < 0.05, **p < 0.01, ***p < 0.001, student *t* test) (magnification × 10).

Since NPM is phosphorylated on Thr234 and Thr237 by cyclin-dependent kinase (CDK) 1/cyclinB [11], we tested whether knockdown of CDK1 had a role in the regulation of p-NPM-Thr^234/237^ mediated HCC metastasis. To examine whether CDK1 is critically involved in p-NPM-Thr^234/237^ mediated migration and invasion, we repressed expression of CDK1 in high p-NPM-Thr^234/237^ expressing cells (SMMC and Bel 7402). By qPCR and western blot analyses, we found successful knockdown of CDK1 (#C10 and #C11) in SMMC and Bel 7402 cells (Figure 4A and 4B). Accompanied with the reduction in CDK1 expression, p-NPM-Thr^234/237^ was consistently suppressed in two shCDK1 clones when compared to NTC control, which suggesting that CDK1 is upstream kinase leading to NPM phosphorylation at Thr234 and Thr237 (Figure 4B). After confirming reduction of CDK1 and p-NPM-Thr^234/237^expression upon CDK1 knockdown, we examined the effect of CDK1 knockdown in cell proliferation. By BrdU assay, suppression of CDK1 significantly reduced cell proliferation in both SMMC and Bel 7402 cells (Figure 4C). Next, we examined the effect of CDK1 knockdown in cell migration and invasion. Accompanied with reduction of p-NPM-Thr^234/237^ expression of by CDK1 knockdown, suppression of CDK1 significantly also reduced the migration and invasion of HCC cells (Figure 4D and 4E). In addition to the knockdown approach, we further examined the effect of CDK1 suppression on HCC cell migration and invasion using CDK1 inhibitor (RO-3306, Sigma). Upon treatment at 5 and 10 μM for 24 hrs, we consistently found decreased number of migrated and invaded cells in Bel 7402 and SMMC cells when compared with untreated control (Supplementary Figure 3).

### p-NPM-Thr^234/237^ binds ROCK2 and regulates LIMK signaling

**Figure 5:**
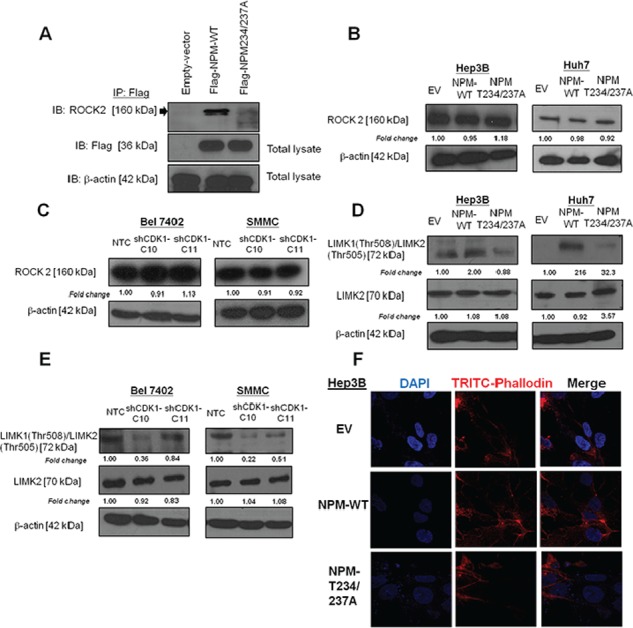
p-NPM-Thr^234/237^ regulated metastasis through Rho-ROCK-LIM kinase pathway **A.** Immunoprecipitation assays revealed a physical interaction between NPM and ROCK2. **B.** The protein expression of ROCK2 in NPM-wild type (NPM WT) and NPM Thr234/237A mutant was comparable to control. **C.** Knockdown of CDK1 did not change the protein expression of ROCK2 when compared to NTC. **D.** NPM Thr234/237A mutate reduced phosphorylation of LIM kinase (LIMK), a substrate of ROCK2, in Hep3B and Huh7 transfected cells. **E.** Similar results were observed when CDK1 was knocked down in Bel 7402 and SMMC cells. **F.** NPM WT transfectants exhibited increased stress fiber network. Stress fibers (polymerized actin) and actin filaments were demonstrated by phalloidin (red), while NPM Thr234/237A mutant exhibited loss of stress fibers, when compared with NPM WT. (magnification × 63).

NPM was found to interact physically with ROCK2 during centrosome duplication, and NPM phosphorylation at Thr^199^ showed enhanced interaction with ROCK2 [10]. In addition, ROCK2 was found to be overexpressed and played critical role in HCC invasiveness [14]. To further examine whether ROCK2 is one of the major downstream effectors of NPM-mediated HCC metastasis, we first confirmed the binding between NPM and ROCK2 by co-transfecting Flag-tagged NPM plasmid and its mutant into Huh7 cells. Immunoprecipitation assays revealed a physical interaction between NPM and ROCK2 (Figure 5A). However, the physical binding is largely diminished when Flag-tagged NPM-Thr234/237A was transfected. The result indicated that defective Thr234/237 phosphorylation decreased the binding affinity for ROCK2 (Figure 5A). Apart from its effect on binding affinity, we examined whether p-NPM-Thr^234/237^ affects ROCK2 protein expression. However, ROCK2 expression did not change upon transfection of Flag-tagged NPM and its phosphorylation site mutant (Thr234/237A) (Figure 5B). Similar result was found in SMMC and Bel 7402 cells transfected with shCDK1 (Figure 5C). To further examine whether this observation is clinically relevant, we examined ROCK2 expression in 28 cases of primary and their matched distinct extra-hepatic metastatic tissues. Consistently, we found no significant difference in ROCK2 expression between primary and their matched extra-hepatic metastatic tissues (Supplementary Figure 4). Next, we would like to examine the effect of p-NPM-Thr^234/237^ on ROCK2 kinase activity. To evaluate this potential effect, we examined the level of phosphorylation of LIMK, a substrate of ROCK2, responsible for actin polymerization and depolymerization. We found increase in LIMK1 (Thr508)/LIMK2 (Thr505) expression in Flag-tagged NPM cells from Huh7 and Hep3B cells, while this effect was abolished in Thr234/237A transfectants (Figure 5D). Consistently, knockdown of CDK1 decreased expression of LIMK1 (Thr508)/LIMK2 (Thr505) (Figure 5E). In addition, we examined the effect of p-NPM-Thr^234/237^ on expression of MMP-2, and EMT markers including E-cadherin, Vimentin and Snail. The result revealed that the expression of these markers in NPM-wild type (NPM WT) and NPM Thr234/237A mutant was comparable to control. Similar result was observed when CDK1 was knocked down in Bel 7402 and SMMC (Supplementary Figure 5). These findings suggest that CDK1-mediated phosphorylation of NPM is crucial in metastasis through regulation of actin polymerization but not EMT and MMP protein. To further investigate whether NPM phosphorylation regulates cell motility and cancer invasion through modulation of actin stress fiber, we examined stress fiber formation and polymerized actin in EV, Flag-tagged NPM and its phosphorylation site mutant (Thr234/237A) of Hep3B cells. Cytoskeletal reorganization exemplified by the formation of stress fiber bundling arrays is essential for the contractile motion of cancer cells. Using phalloidin staining, we found that stress fiber formation was enhanced in NPM stable transfectants, while its effect was abolished in its phosphorylation site mutant (Thr234/237A) (Figure 5F).

## DISCUSSION

Several chromosomal alterations, such as 13q [15] and amplification of 1q12-q22 [16, 17], have been reported in HCC metastasis. The advent of cDNA microarray technology allows the use of genome-wide expression profiling to identify metastasis-associated genes [18]. It is now generally recognized that mRNA microarray results should be considered preliminary data that require independent follow-up validation because proteins, rather than transcripts, are the major effectors of cellular function [19]. Therefore, the identification of proteins critical for metastatic process by proteomic profiling has become a new avenue to study HCC metastasis. However, proteomic analyses are complicated by post-translational modifications to proteins. One such modification, protein phosphorylation, plays a significant role in many physiological processes including HCC metastasis [20]. Using a pair of primary and a matched metastatic counterpart (PLC/PRF-PT and PLC/PRF/5-LM), we have compared their phosphorylation profiles and identified p-NPM-Thr^234/237^ to be upregulated in PLC/PRF/5-LM. Such observation was also observed in another pair of primary and a matched metastatic counterpart (MHCC-97L and MHCC-97H), suggesting p-NPM-Thr^234/237^ upregulation in metastatic HCC cell line is not cell line specific.

The physiological function of NPM in tumorigenesis remains controversial. NPM mutant mice have aberrant oncogenesis, which leads to unrestricted genomic instability [4]. In contrast, mutations in NPM in acute myelogenous leukemia disrupt the NPM nucleolar-localization signal, causing accumulation of NPM in the cytoplasm [21]. Moreover, NPM may contribute to oncogenesis by activating the oncogenic potential of a fused protein partner, such as RARα [22]. In HCC, NPM overexpression was found to be associated with pathological grading [22], metastatic behavior [23, 24] and drug resistance [23, 24]. Despite all these findings, how phosphorylation events of NPM affects the tumor behavior has not been addressed so far. Our results suggested that p-NPM-Thr^234/237^ is critical in invasion and migration of HCC cells. Since NPM is phosphorylated on Thr234 and Thr237 by cyclin-dependent kinase (CDK) 1 [11], we tested whether knockdown of CDK1 had a role in the regulation of p-NPM-Thr^234/237^ mediated HCC metastasis. Similar to the effect to the Thr234/237A transfectants, knockdown of CDK1 also suppressed HCC cell proliferation, migration, and invasion. Our result is consistent to previous report showing that CDK1 is overexpressed in HCC and its expression is correlated with aggressive tumor behavior and recurrence of HCC [25]. In addition, CDK1 was reported to be a useful biomarker for HCC to distinguish from non-tumor liver [26]. Recently, CDK1 is an attractive therapeutic target for suppression of HCC metastasis by a garlic derivative (SAC) [27].

NPM was found to interact physically with ROCK2 during centrosome duplication, and NPM phosphorylation at Thr^199^ showed enhanced interaction with ROCK2 [10]. ROCK2 was found to be overexpressed and played critical role in HCC invasiveness through regulation of actin polymerization [14], MMP2 degradation [28] and EMT [29]. To further examine whether ROCK2 is one of the major downstream effectors of NPM-mediated HCC metastasis, we first confirmed the binding between NPM and ROCK2 by transfecting flag-tagged NPM plasmids into Huh7 cells. To explore whether p-NPM-Thr^234/237^ is critical for NPM/ROCK2 complex formation, we performed immunoprecipitation after transfection of Flag-tagged Thr234/237A into Huh7 cells. The result indicated that defective in Thr234/237 phosphorylation decreased the binding affinity for ROCK2, when compared with the wild-type NPM. By western blot analysis, we found that such alteration of binding between NPM and ROCK2 did not affect the protein expression of ROCK2. Instead, it affected the transcriptional activity of ROCK2, which resulted in change in expression of its downstream transcriptional target LIMK1/LIMK2. ROCK2/LIMK/cofilin pathway was previously reported to maintain actin stress fibers in fibroblasts [30]. Consistently, we found p-NPM-Thr^234/237^ regulates phosphorylation of LIMK, which is accompanied with increase in stress fiber formation.

In conclusion, we found that CDK1 mediated phosphorylation of p-NPM-Thr^234/237^ may play a role in growth and metastasis of HCC cells through interaction with a novel metastatic gene, ROCK2. Thus, targeting of CDK1/p-NPM/ROCK2 signaling pathway may be a potential therapeutic strategy for treatment of HCC patients.

## MATERIALS AND METHODS

### Cell lines

The human HCC cell lines Huh7, HLE, PLC/PRF/5 (Japanese Cancer Research Bank, Japan), Bel 7402, SMMC-7721, (SIBS, Chinese Academy of Sciences), Hep3B, MHCC-97L, MHCC-97H, (Liver Cancer Institute, Fudan University, China) were grown in Dubecco's modified Eagle minimal high glucose essential medium (DMEM-HG) (Gibco-BRL) supplemented with 10% fetal bovine serum (FBS) (Gibco-BRL) and 1% penicillin and streptomycin (Gibco-BRL).

### Human HCC samples

Human primary HCCs and their metastatic counterparts, and corresponding non-tumorous liver samples in the cohort of 28 primary HCCs were obtained from patients with liver resection between 1993 and 2007 at Queen Mary Hospital, Hong Kong and randomly selected for the present study. The metastatic sites of these 28 HCC cases was shown in Table 1. All specimens collected were either snap-frozen in liquid nitrogen and stored at −80°C, or fixed in 10% formalin for paraffin embedding. Frozen sections from tumorous and non-tumorous liver samples were cut and stained for histological examination to ensure homogenous cell population of tissues. Use of human specimens was approved by the institutional review board of the University of Hong Kong/Hospital Authority Hong Kong West Cluster.

**Table 1:** Sites of extrahepatic metastasis in 28 primary HCC samples

Case number	Sites of extrahepatic metastasis
1	Intraperitoneal tissue
2	Diaphragm
3	Main portal vein thrombus
4	Lung
5	Adrenal
6	Bone
7	Brain
8	Peritoneum
9	Adrenal
10	Lung
12	Spleen
13	Colon
16	Spinal
17	Abdominal wall
20	Lymph node
21	Thoracic cavity
22	Retro-caval lymph node
23	Scapular nodule
24	Colon
25	Lung
26	Lymph node
27	Diaphragm
28	Spleen
29	Bone
30	Retro-pancreatic tissue
31	Brain
33	Omentum
36	Lung

### Plasmids and cloning

The full-length human cDNA (HA-NPM) and its NPM mutants containing threonine to alanine mutations at Thr^234^ and Thr^237^ (HA-NPM-Thr234/237) were a gift from Dr. Paul Shapiro (University of Maryland, USA). Flag-tagged -NPM and -NPM-Thr234/237A were further subcloned to lentivial based pCDH vector (System Biosciences). The sequence of NPM and NPM-Thr234/237 were verified by DNA sequencing.

### CelluSpot™ serine/threonine kinase I peptide array

CelluSpots™ Serine/Threonine kinase I peptide array was purchased from Intavis AG (Köln, Germany). Each slide contains 384 evaluated kinase substrates as well as consensus sequences for serine/threonine kinases spotted in duplicate.

### Binding of protein lysate to peptide array

Protein lysate of PLC/PRF/5-PT and PLC/PRF/5-LM was prepared by solubilizing their corresponding cell pellets in cell lysis buffer following by sonication. After determination of the total protein concentration, 300 μg/ml of protein lysate was added to cell lysate reaction mixture containing 10 μM ATP and 300 μCi/mL[γ-33^P^]ATP and incubated with incubation chamber for 2 hours. After incubation, the slide was washed two times 5 minutes with washing buffer, followed by three washing steps in dH_2_O for 5 minutes at room temperature. The dried slide was imaged on x-ray film for different time periods. The intensity of phosphorylation profiles was evaluated by gel documentation system.

### Lentiviral-based transfection into HCC cells

For suppression of NPM in HCC cells, lentiviral particles (DFCI-Broad RNAi Consortium, Boston) expressing shRNAs against human NPM were used to downregulate *NPM* mRNA (CCGGGCCAAGAATGTGT TGTCCAAACTCGAGTT TGGACAACACATTCTTGG CTTTTTG). For suppression of CDK1 in HCC cells, lentiviral particles (DFCI-Broad RNAi Consortium, Boston) expressing shRNAs against human CDK1 were used to downregulate *CDK1* mRNA (clone C10:CCGGGCTGTACTTCGTCTTCTAATTCTCGAGAATTAGAAGACGAAGTACAGCTTTTT; clone C11:CCGGGTGGAATCTTTACAGGACTATCTCGAGATAGTCCTGTAAAGATTCCACTTTTT). For functional characterization of NPM and its phosphorylation mutant, we have ectopically transfected flag-tagged NPM or its phosphorylation site mutants (Thr234/237A) by the lentiviral based approach provided by System Biosciences. Transduced cells were selected with 2 μg/mL puromycin.

### Proliferation assay

Cells at the density of 5 × 10^3^ were seeded in 96-well cell culture plates and allowed to adhere overnight. At the indicated time points, cell proliferation was measured by using BrdU proliferation assay kit (Roche Diagnostics Corporation, Indianapolis, Indiana, USA) according to manufacturer's instructions. The experiment was carried out thrice independently.

### Migration and invasion assays

Cell migration was investigated using transwell inserts with 6.5 mm polucarboate membranes of 8.0 μm pore size (Corning Inc., NY, USA). Cells (1*e5) in serum-free medium were seeded onto the upper chamber of the transwell and the culture medium containing 10% FBS was added into the lower chamber serving as chemoattractant. Cells were incubated for 24 hr to allow for migration through the membrane. Migrated cells were then fixed by 2% paraformaldehyde and stained with crystal violet. Stained cells were captured randomly for three fields under a light microscope and ells were counted. The experiments were repeated independently three times.

The cell invasion assay was performed with self-coated Matrigel (BD Biosciences, San Jose, CA) on the upper surface of a transwell chamber. The invasive cells that had invaded through the extracellular matrix layer to the lower surface of the membrane were fixed with 2% paraformaldehyde and stained with crystal violet. Photographs of three randomly selected fields of the fixed cells were captured and cells were counted. The experiments were repeated independently three times.

### *In vitro* scratch assay

Cells were seeded in 12-well plates and culture till confluent. P20 pipette tip was used to create a scratch of the cell monolayer. The plate was washed once and replaced with the desired medium. Images were captured (magnification × 4) at 0 hr and 24 hr.

### Quantitative PCR (qPCR) analysis

Total RNA was isolated using Trizol reagent according to the manufacturer's protocol (Invitrogen, Carlsbad, CA). Complementary DNA (cDNA) was synthesized using a GeneAmp® Gold RNA PCR Kit (Applied Biosystems, Foster City, CA) according to the manufacturer's instructions and then subjected to PCR with a SYBR Green PCR kit. The primer sequence for CDK1 is (Forward: 5′-CTGGGGTCAGCTCGTTACTC-3′; Reverse: 5′-TCCACTTCTGGCCACACTTC-3)′. The amplification protocol consisted of incubations at 94°C for 15 seconds, 63°C for 30 seconds, and 72°C for 60 seconds. Incorporation of the SYBR Green dye into PCR products was monitored in real time with an ABI 7900HT Sequence Detection System and SDS 1.9.1 software (Applied Biosystems) and subsequently analyzed using RQ Manager 1.2 software (Applied Biosystems), thereby allowing the threshold cycle (*C*_T_) at which exponential amplification of the products began to be determined. The amount of target cDNA was calculated relative to that of β-actin cDNA.

### Western blot analysis

Western blots were developed using an ECL Plus kit (Amersham Biosciences, Piscataway, NJ). The primary antibodies included rabbit polyclonal anti-CDK1/CDC2 (Proteintech), rabbit monoclonal anti-LIMK2 (Cell signaling technology, Danvers, MA, USA), rabbit polyclonal anti-phospho-LIMK1 (Thr508) / LIMK2 (Thr505) (Cell signaling technology, Danvers, MA, USA), mouse monoclonal anti-NPM (Invitrogen Corporation, CA), rabbit polyclonal anti-phosphorylated Thr234/Thr237 NPM (BioLegend, CA), rabbit polyclonal anti-ROCK2 (Santa Cruz Biotechnology, CA), mouse polyclonal anti-Actin (Sigma-Aldrich, St. Louis, MO, USA). After washing, the membrane was incubated with horseradish peroxidase-conjugated anti-mouse or rabbit antibody (Amersham) and then visualized with enhanced chemiluminescence plus according to the manufacturer's protocol.

### Immunoprecipitation

Cells transiently transfected with different constructs were lysed with RIPA buffer. The lysate was then precleared with 30 μl of protein G agarose (Amersham, Piscataway, NJ) and normal mouse IgG for 1 h at 4°C. The precleared lysate was then incubated with anti-Flag (Sigma-Aldrich, St. Louis, MO, USA) antibody for 1 h at 4°C and then with protein G agarose for another hour at 4°C. The agarose was then washed three times with 1 ml of RIPA buffer, boiled and then loaded onto SDS-polyacrylamide gel for electrophoresis and Western blotting analysis with the procedure described above.

### Immunohistochemical staining

For paraffin-embedded tissues, sections were deparaffinized in xylene and rehydrated in graded alcohols and distilled water. Slides were processed for antigen retrieval by a standard microwave heating technique. Specimens were incubated with NPM (B0556, Sigma), p-NPM-Thr^234/237^ (619102, Biolegend) and ROCK2 (21645-1-AP, Proteintech) in a dilution of 1:100. Subsequent immunodetection was performed using the standard rapid EnVision technique. The reaction was then developed with the DAKO Liquid DAB_Substrate-Chromogen System (DAKO, Carpentaria, CA). Sections were counterstained with Mayer's hematoxylin. Stained slides were imaged on an Aperio Scanscope® CS imager (Vista, CA), generating 0.43 μm/pixel whole slide images. These images were compiled, and NPM and p-NPM-Thr^234/237^ expression in terms of intensity and abundance were quantitated using the Aperio Spectrum® software with a pixel count algorithm.

### Immunofluorescence microscopy

Cells were seeded onto coverslips and incubated overnight at 37°C in a CO2 incubator, then were fixed with 4% paraformaldehyde in phosphate-buffered saline (PBS) and permeabilized with 0.1% Triton X-100 in PBS. Fixed cells were incubated with 1:5000 tetramethylrhodamine-conjugated phalloidin (Sigma-Aldrich, St. Louis, MO, USA). Cells were counterstained with 4′,6-diamidino-2-phenylindole (DAPI) (Calbiochem, San Diego, CA) and mounted with Vectashield antifade mountant (Vector Laboratories, Burlingame, CA). Images were captured under × 63 magnification by a fluorescence microscope (Carl Zeiss LSM 510 Meta/Axiocam).

### Statistical analysis

All statistical analyses were performed using the statistical software SPSS 17 for Windows (SPSS Inc., Chicago, IL). Student's *t* was used for continuous data wherever appropriate. *p* values less than 0.05 were considered significant.

## SUPPLEMENTARY MATERIAL FIGURES AND TABLE





## Abbreviations

CDK1: cyclin dependent kinase 1
HCC: hepatocellular carcinoma
NPM: nucleophosmin
LIMK: LIM kinase
IHC: immunohistochemistry
ROCK2: Rho kinase II
