# Supplementary material for: Phosphorylation of Nucleophosmin at Threonine 234/237 is associated with HCC metastasis
Source: Oncotarget. 2015 Oct 30;6(41):43483–95. doi: 10.18632/oncotarget.5820 (PMC4791245; doi:10.18632/oncotarget.5820)
Supplement: Supplementary file 1 [file oncotarget-06-43483-s001.pdf]

SUPPLEMENTARY FIGURES AND TABLE

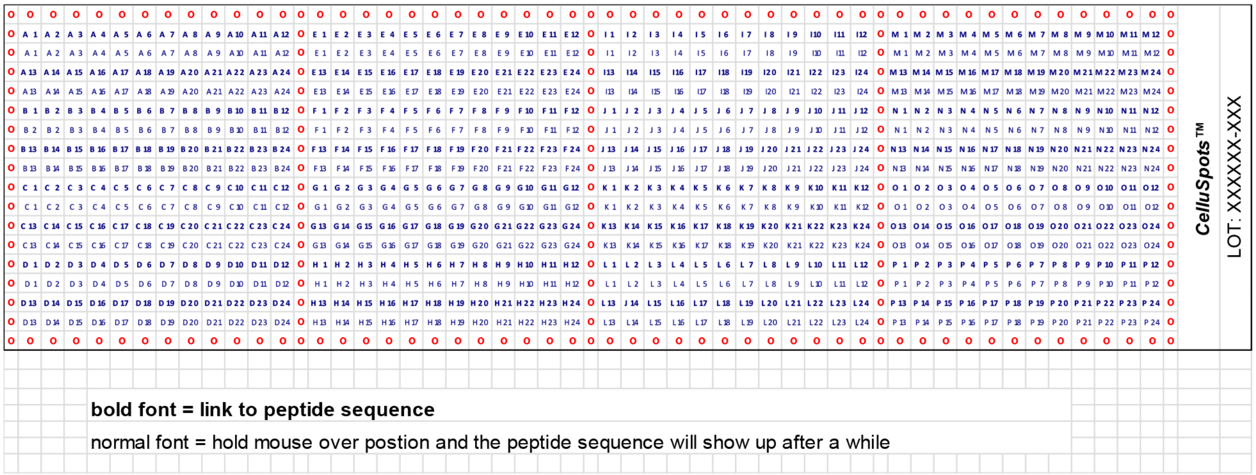

Supplementary Figure S1: The plate set up of CelluSpot™ Serine/Threonine kinase I peptide array. The glass based array consists of 384 spots in duplicates and each spot consists of 15-mer peptide (substrate) bound to cellulose.

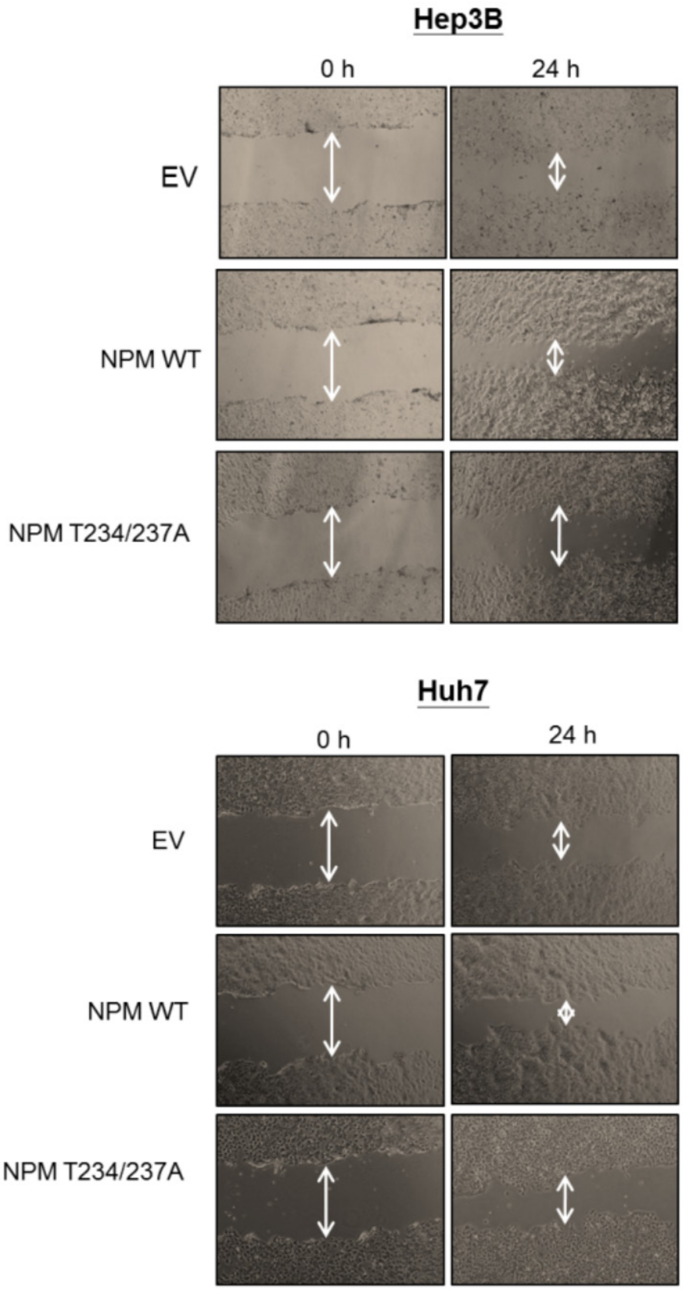

Supplementary Figure S2: p-NPM-Thr234/237 enhanced HCC cell migration and invasion by using *in vitro* scratch assay. NPM wild-type transfected Hep3B and Huh7 cells (NPM WT) enhanced migratory ability when compared with respective control (EV) and NPM T234/237 mutant (NPM Thr234/237A). Images were acquired at 0 and 24 h in *in vitro* scratch assay.

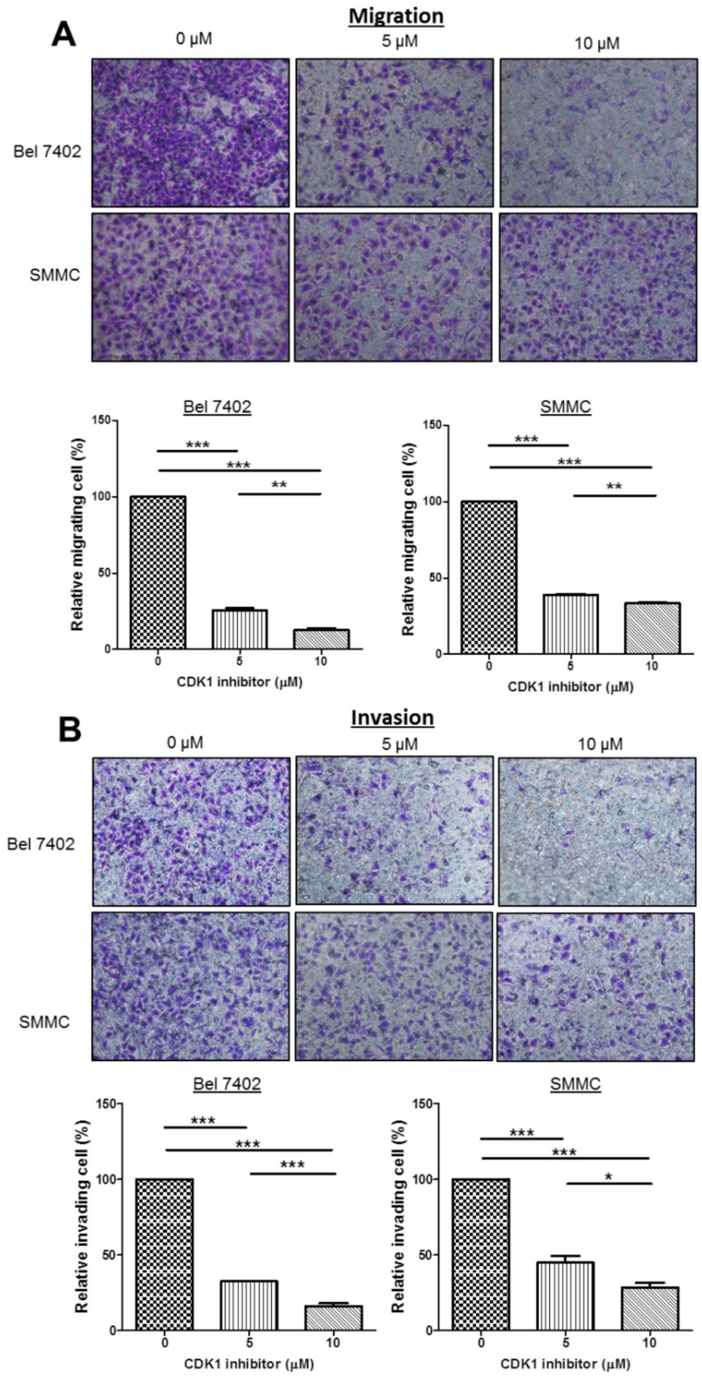

Supplementary Figure S3: CDK inhibition reduced migratory and invasive abilities of HCC cells. Upon CDK1 inhibitor (Sigma, RO-3306) treatment, the number of **A.** migrated and **B.** invaded cells was significantly lower in Bel 7402 and SMMC cells when compared with untreated control (*$p < 0.05$, **$p < 0.01$, ***$p < 0.001$, student t test).

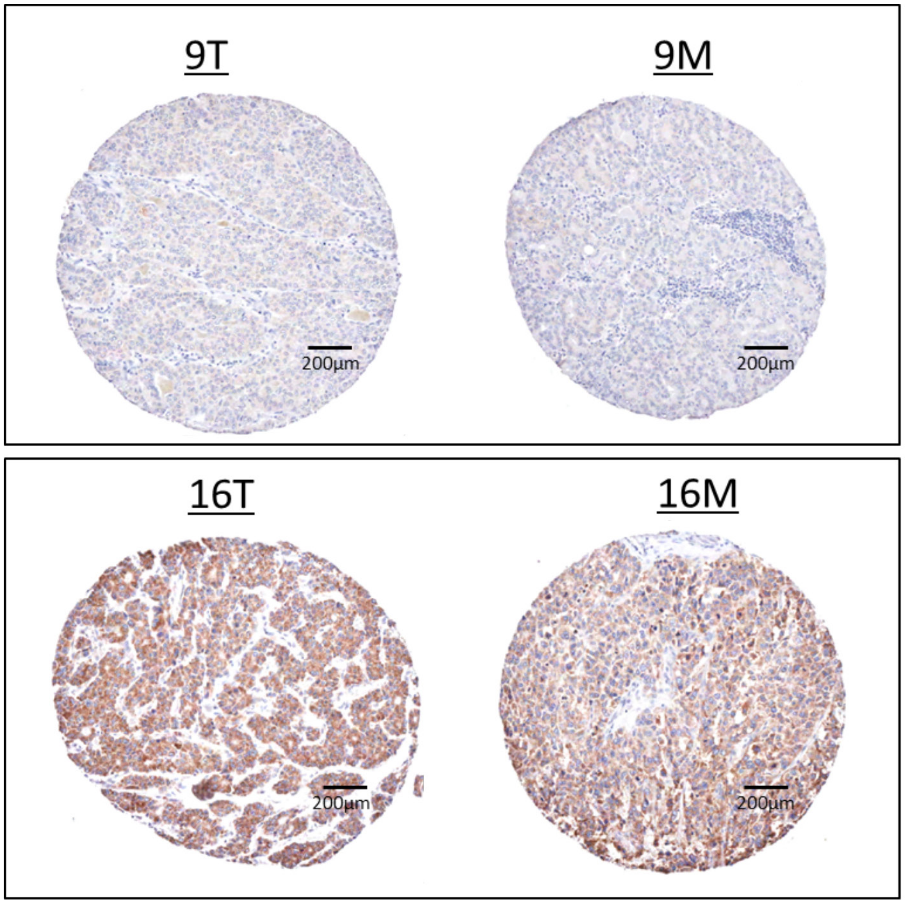

No of cases (T>M)	No of cases (T=M)	No of cases (T<M)
7	19	2

Supplementary Figure S4: ROCK 2 expression in primary and matched metastatic HCCs. In 28 cases primary and matched distinct metastatic tissues, immunohistochemistry revealed no significant difference between primary and metastatic HCCs for ROCK2 expression. Two representative cases (case #9 (adrenal metastasis) and case #16 (spinal metastasis)) were shown.

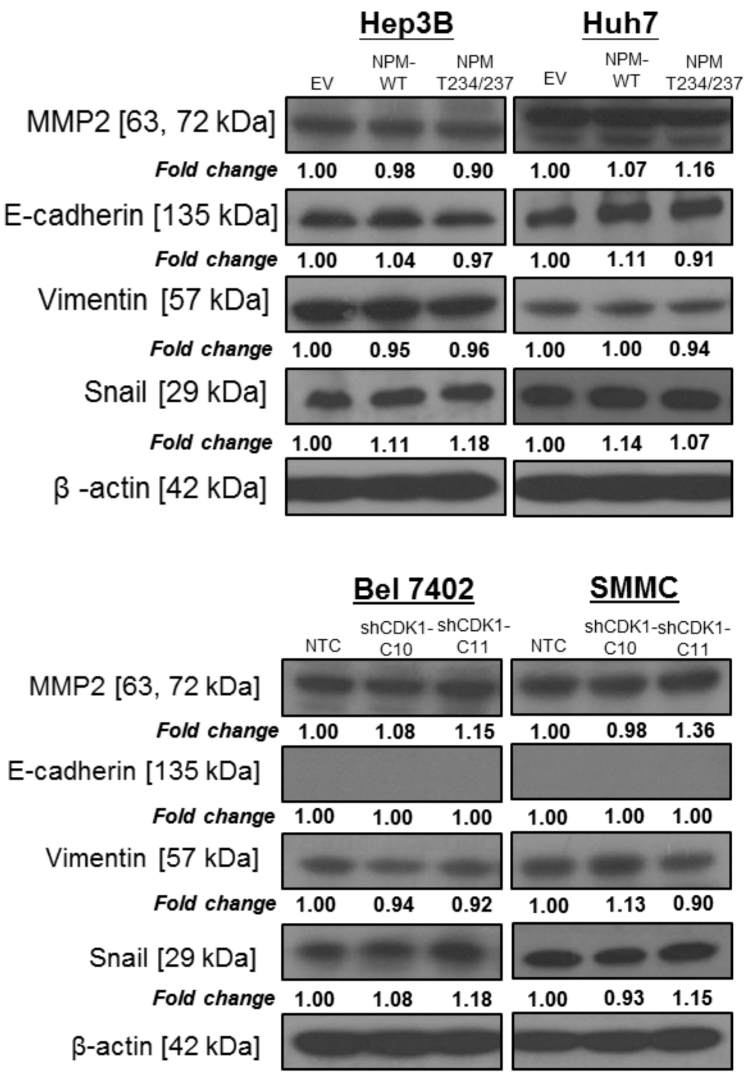

Supplementary Figure S5: Effect of p-NPM-Thr234/237 on expression of EMT markers and invasiveness markers. A. Immunoblotting demonstrated that the protein expression of an invasiveness marker, MMP2, and a panel of EMT markers in NPM-wild type (NPM WT) and NPM Thr234/237A mutant was comparable to control. **B.** Knockdown of CDK1 did not alter the protein expression of MMP2 and EMT markers when compared with control.

Supplementary Table S1: A list of peptide sequences of kinases included in CelluSpot™ Serine/Threonine kinase I peptide array. Each slide contains 384 evaluated kinase substrates as well as consensus sequences for serine/threonine kinases spotted in duplicate. The peptide sequences of kinases with fold difference [LM/PM] > 2 are specified in the table.
