# Supplementary material for: Phosphorylation of Nucleophosmin at Threonine 234/237 is associated with HCC metastasis
Source: Oncotarget. 2015 Oct 30;6(41):43483–95. doi: 10.18632/oncotarget.5820 (PMC4791245; doi:10.18632/oncotarget.5820)
Supplement: Supplementary file 2 [file oncotarget-06-43483-s002.docx]

**Supplementary Tables**

**Supplementary Table 1. A list of peptide sequences of kinases included in CelluSpot™ Serine/Threonine kinase I peptide array.** Each slide contains 384 evaluated kinase substrates as well as consensus sequences for serine/threonine kinases spotted in duplicate. The peptide sequences of kinases with fold difference [LM/PM] > 2 are specified in the table.

| ***Well*** | ***Kinase*** | ***Substrate*** | ***Position*** | ***Fold Change [LM/PM] Fold Change >2*** |
| --- | --- | --- | --- | --- |
| D24 | CDK1 | NPM | T234 | **4.51** |
| B13 | AuroraBkinase | Kinesincentralmotor1 | T95 | **4.24** |
| D19 | CDK1 | HistoneacetyltransferaseHTATIP | S86 | **4.19** |
| A13 | AMPK_group |  | ST11 | **4.16** |
| A 1 | control |  |  | **4.13** |
| D21 | CDK1 | HMGI-C | S44 | **4.07** |
| A14 | ATM | Abl | S446 | **4.03** |
| B 7 | AuroraAkinase |  | ST11 | **3.98** |
| A23 | ATM | Rad9 | S272 | **3.95** |
| P10 | MLCK | MLCK | T289 | **3.95** |
| P11 | MLCK | Myosinregulatorylightchain2,skeletalmuscle | S15 | **3.79** |
| G17 | CK2 | Insulinreceptorsubstrate1 | S24 | **3.72** |
| J11 | GSK-3_group | CREB | S129 | **3.70** |
| O 9 | MAPK14 | RibosomalproteinS6kinasealpha4 | S196 | **3.66** |
| P22 | NLK |  | S166 | **3.64** |
| G14 | CK2 | n-myc | S261 | **3.62** |
| K12 | ILK | CPI-17 | T38 | **3.60** |
| P19 | NEK6 |  | ST11 | **3.58** |
| I 4 | ERA | ERA | T36 | **3.58** |
| P 6 | MFPK |  | S450 | **3.56** |
| P18 | NEK6 | RibosomalproteinS6kinase1 | S53 | **3.56** |
| O24 | MAPKAPK2 | Arachidonate5lipoxygenase | S271 | **3.55** |
| O10 | MAPK14 | Sodium/hydrogenexchanger1 | T718 | **3.55** |
| I 3 | Eg3kinase | cdc25B | S353 | **3.55** |
| D13 | CDK1 | Cytokeratin18 | S33 | **3.52** |
| O22 | MAPKAPK2 | Lymphocytespecificprotein | S204 | **3.52** |
| H 1 | CK2 | HOXB7 | S133 | **3.50** |
| N23 | MAPK10 |  | S353 | **3.50** |
| O 8 | MAPK14 | PAK6 | S165 | **3.47** |
| P 9 | MHCK |  | ST11 | **3.47** |
| P 7 | MHCK | MyosinIC | S311 | **3.44** |
| O21 | MAPKAPK2 | Tuberin | S1254 | **3.43** |
| K10 | IKK-beta | NF-kappa-Bessentialmodulator | S31 | **3.43** |
| K22 | IPL1 | Hypothetical80.5kDaproteininSLN1-RAD25intergenicregion | S100 | **3.41** |
| P12 | MLCK |  | ST11 | **3.40** |
| A11 | AMPK_group | eEF-2kinase | S78 | **3.40** |
| H 2 | CK2 | Beta-arrestin2 | T382 | **3.38** |
| G22 | CK2 | TFIIA42 | S280 | **3.37** |
| O12 | MAPK14 |  | ST11 | **3.36** |
| K 8 | IKK_group |  | ST11 | **3.35** |
| G20 | CK2 | Neutrophilcytosolfactor1 | S208 | **3.35** |
| K13 | ILK | Proteinphosphatase1regulatorysubunit14B | T57 | **3.34** |
| E15 | CDK2 | UBF-1 | S389 | **3.32** |
| P16 | NEK2 | Serine/threonineproteinphosphatasePP1-gammacatalyticsubunit | T307 | **3.31** |
| A 2 | neg. control |  |  | **3.31** |
| H17 | DAPK3 | Mdm2 | S166 | **3.30** |
| G18 | CK2 | CD45 | S999 | **3.30** |
| O11 | MAPK14 | TAK1-bindingprotein1 | S423 | **3.30** |
| H14 | DAPK3 | HistoneH3.1 | T11 | **3.29** |
| K11 | IKK-beta |  | ST11 | **3.29** |
| E18 | CDK2 |  | ST11 | **3.28** |
| E17 | CDK2 | Coilin | S184 | **3.28** |
| K 9 | IKK-beta | p65 | S536 | **3.27** |
| F23 | CK1delta |  | S329 | **3.26** |
| H10 | CSFR1 | CSFR-1 | S560 | **3.26** |
| G19 | CK2 | RAD1 | S214 | **3.26** |
| G 1 | CK1_group | APC | S1279 | **3.26** |
| P23 | control |  |  | **3.25** |
| I14 | GRK-1 | Rhodopsin | S334 | **3.25** |
| P 5 | MARK_group |  | S1073 | **3.24** |
| P14 | NEK | Phospholemmanprecursor | S83 | **3.24** |
| O23 | MAPKAPK2 | Tristetraproline | S52 | **3.24** |
| E16 | CDK2 | Celldivisioncontrolprotein6homolog | S54 | **3.23** |
| H19 | DAPK3 |  | ST11 | **3.23** |
| N15 | MAPK1 | Vinexin | S594 | **3.22** |
| H18 | DAPK3 | p21 | T144 | **3.22** |
| I22 | GRK-2 | Tubulinbeta-3chain | T409 | **3.22** |
| K 5 | IKK_group | IRF3 | S386 | **3.21** |
| H16 | DAPK3 | CPI-17 | S12 | **3.20** |
| N 7 | MAPK_group | p53 | T55 | **3.20** |
| H23 | DNA-PK | DNA-repairproteinXRCC4 | S193 | **3.18** |
| I15 | GRK-1 |  | ST11 | **3.18** |
| K 6 | IKK_group | NF-kappa-Bessentialmodulator | S369 | **3.18** |
| K21 | IPL1 | Innercentromereprotein | S578 | **3.18** |
| P24 | neg. control |  |  | **3.17** |
| G21 | CK2 | TFIIAalphaandbetalikefactor | S356 | **3.15** |
| G16 | CK2 | G6Pisomerase | S184 | **3.15** |
| H 9 | CK2alpha | eIF-5 | S174 | **3.14** |
| F21 | CK |  | ST11 | **3.13** |
| N22 | MAPK10 | Bcl-2-likeprotein11 | S58 | **3.13** |
| P21 | NLK | LEF-1 | T155 | **3.12** |
| A 7 | AMPK_group | eEF-2kinase | S366 | **3.12** |
| O 1 | MAPK11 | HBP1 | S402 | **3.12** |
| I19 | GRK-2 | FMLPreceptor | S328 | **3.11** |
| N 6 | MAPK_group | p21 | T56 | **3.11** |
| A20 | ATM | E2F-1 | S31 | **3.11** |
| B 2 | ATR |  | S345 | **3.11** |
| N16 | MAPK1 | Fos | T232 | **3.11** |
| H11 | DAPK_group | Calcium/calmodulin-dependentproteinkinasekinase2 | S511 | **3.10** |
| G13 | CK2 | HSP90-beta | S225 | **3.09** |
| D 6 | CDK | Retinoblastomalike2 | S413 | **3.09** |
| J10 | GSK-3_group | ATP-citratesynthase | T447 | **3.09** |
| D 7 | CDK | Src | S74 | **3.08** |
| E 3 | CDK1 | Plectin1 | T4539 | **3.08** |
| O 5 | MAPK13 | eEF-2kinase | S359 | **3.08** |
| J 7 | GSK-3_group | eIF-2B | S535 | **3.07** |
| G 5 | CK1_group | IPP2 | S86 | **3.07** |
| K19 | IPL1 | DASHcomplexsubunitASK1 | S26 | **3.07** |
| H20 | DNA-PK | ATP-dependentDNAhelicaseII,80kDasubunit | S577 | **3.07** |
| B 8 | AuroraBkinase | AIM-1 | T232 | **3.07** |
| E 4 | CDK1 | Rap1GAP | S484 | **3.07** |
| I18 | GRK-2 | Delta-typeopioidreceptor | T358 | **3.06** |
| E 1 | CDK1 | Originrecognitioncomplexsubunit1 | S258 | **3.06** |
| I24 | GRK-3 | Mu-typeopioidreceptor | T180 | **3.06** |
| H 8 | CK2 |  | ST11 | **3.05** |
| F20 | CK | Band3aniontransportprotein | S29 | **3.05** |
| D23 | CDK1 | Neuralinterleukin16protein | T757 | **3.05** |
| A19 | ATM | DoublestrandbreakrepairproteinMRE11A | S264 | **3.04** |
| O 3 | MAPK12 |  | S201 | **3.04** |
| E19 | CDK4 | Retinoblastomalike1 | T369 | **3.04** |
| E21 | CDK4 | Retinoblastomalike2 | T401 | **3.03** |
| J23 | GSK-3beta |  | ST11 | **3.03** |
| K14 | ILK | ILK-1 | S343 | **3.03** |
| I21 | GRK-2 | Tubulinbeta-3chain | S444 | **3.02** |
| N21 | MAPK10 |  | S73 | **3.02** |
| I16 | GRK-2 | 60SacidicribosomalproteinP2 | S102 | **3.02** |
| G15 | CK2 | Estrogenreceptoralpha | S167 | **3.02** |
| N 5 | MAPK_group | Myocyte-specificenhancerfactor2C | S387 | **3.01** |
| I 7 | ERTPK | c-myc | S62 | **3.01** |
| H 4 | CK2 | DNAligaseI | S66 | **3.01** |
| K16 | ILK | PKB-alpha | S473 | **3.00** |
| I23 | GRK-2 |  | ST11 | **3.00** |
| C13 | CCDPK | CaMKI-alpha | T177 | **3.00** |
| N19 | MAPK1 |  | ST11 | **2.99** |
| G 8 | CK1_group |  | ST11 | **2.99** |
| N20 | MAPK10 | Bcl-2-likeprotein11 | S55 | **2.99** |
| N14 | MAPK1 | Bcl-2-likeprotein11 | S69 | **2.99** |
| D18 | CDK1 | Hamartin | T417 | **2.99** |
| A 8 | AMPK_group | Lipasehormonesensitive | S400 | **2.99** |
| I13 | GRK_group |  | ST11 | **2.99** |
| E20 | CDK4 | UBF-1 | S484 | **2.98** |
| H12 | DAPK_group | DAPkinase1 | S308 | **2.98** |
| O15 | MAPK7 |  | S355 | **2.97** |
| J 5 | GRK-6 | NHERF-1 | S290 | **2.97** |
| D22 | CDK1 | Microtubule-associatedprotein4 | S696 | **2.95** |
| F11 | CHK1 | cdc25A | S124 | **2.95** |
| L 1 | JNK_group | Bcl-2-likeprotein11 | S104 | **2.95** |
| F 9 | CDPK | Nodulin-26 | S262 | **2.95** |
| P 4 | MARK_group | Microtubule-associatedprotein4 | S941 | **2.95** |
| A21 | ATM | Mdm2 | S395 | **2.94** |
| H13 | DAPK_group | Syntaxin1A | S188 | **2.94** |
| D20 | CDK1 | HMG-I(Y) | T52 | **2.94** |
| H 3 | CK2 | PTEN | T366 | **2.93** |
| J 2 | GRK-5 | GRK5 | S484 | **2.92** |
| O 7 | MAPK14 | Myocyte-specificenhancerfactor2A | T312 | **2.92** |
| O17 | MAPK8 | Bcl-2-likeprotein11 | S44 | **2.92** |
| H15 | DAPK3 | Myosinregulatorylightchain2,smoothmuscleisoform | T18 | **2.91** |
| B 3 | AuroraAkinase | TPX2protein | S48 | **2.91** |
| N24 | MAPK10 |  | ST11 | **2.91** |
| B20 | CaM-KII_group | C/EBPbeta | S325 | **2.91** |
| J12 | GSK-3_group | CyclinD1 | T286 | **2.91** |
| G 9 | CK2 | Myogenicfactor5 | S49 | **2.90** |
| G10 | CK2 | Caveolin-1 | S87 | **2.90** |
| I 8 | ERTPK |  | ST11 | **2.90** |
| C10 | CCDPK | Phospholamban | T17 | **2.90** |
| I17 | GRK-2 | Beta-2adrenergicreceptor | T384 | **2.90** |
| K17 | ILK |  | ST11 | **2.90** |
| H 7 | CK2 | Proteasomesubunit,alphatype3 | S242 | **2.90** |
| C22 | CDK | HCDH1 | S151 | **2.89** |
| E23 | CDK5 | Myocyte-specificenhancerfactor2A | S408 | **2.89** |
| B 1 | ATM |  | ST11 | **2.88** |
| F19 | CK | Complementcomponent3 | T1031 | **2.88** |
| J16 | GSK-3beta | AmyloidbetaA4protein | T743 | **2.87** |
| M14 | MAP3K7 |  | ST11 | **2.87** |
| C 2 | CaM-KII_group | Potassiumvoltage-gatedchannelsubfamilyDmember2 | S438 | **2.87** |
| C14 | CCDPK |  | ST11 | **2.87** |
| K18 | IPL1 | DASHcomplexsubunitSPC34 | T199 | **2.87** |
| J 4 | GRK-6 | LeukotrieneB4receptor1 | T308 | **2.87** |
| E22 | CDK4 |  | ST11 | **2.85** |
| K 4 | IKK_group | Insulinreceptorsubstrate2 | S304 | **2.85** |
| N13 | MAPK_group |  | ST11 | **2.85** |
| D 8 | CDK | tau | S518 | **2.84** |
| B11 | AuroraBkinase | HistoneH3.1 | S28 | **2.84** |
| G23 | CK2 | YL-1protein | S41 | **2.84** |
| F 4 | CDK7 | Octamerbindingtranscriptionfactor1 | S385 | **2.84** |
| H 6 | CK2 | Beta-catenin | T102 | **2.83** |
| C11 | CCDPK | Sepiapterinreductase | S213 | **2.83** |
| N17 | MAPK1 | SREBP1 | S117 | **2.83** |
| I20 | GRK-2 | GMP-PDEgamma | T62 | **2.83** |
| J21 | GSK-3beta | Notch2 | T2066 | **2.83** |
| C 3 | CaM-KII_group | SERCA2 | S38 | **2.83** |
| A10 | AMPK_group | 6-phosphofructo-2-kinase/fructose-2,6-biphosphatase2 | S466 | **2.82** |
| D16 | CDK1 | Flapendonuclease-1 | S187 | **2.81** |
| L 9 | MAP2K_group | Sam68 | S58 | **2.81** |
| A 6 | AMPK_group | Raf1 | S259 | **2.81** |
| J19 | GSK-3beta | Microphthalmia-associatedtranscriptionfactor | S401 | **2.81** |
| D10 | CDK1 | Apoptosisinhibitorsurvivin | T34 | **2.80** |
| M13 | MAP3K7 | MAP3K7 | T187 | **2.80** |
| L10 | MAP2K_group | STAT4 | S721 | **2.80** |
| B14 | AuroraBkinase | Myosinregulatorylightchain2,smoothmuscleisoform | S19 | **2.79** |
| K 3 | IKK_group | Insulinreceptorsubstrate1 | S312 | **2.79** |
| A 9 | AMPK_group | AMPKalpha1 | T172 | **2.79** |
| I 6 | ERTPK | c-Jun | S243 | **2.79** |
| F22 | CK1delta | Connexin43 | S324 | **2.78** |
| F10 | CHK1 | p73 | S47 | **2.78** |
| G11 | CK2 | Ref1 | S289 | **2.78** |
| O 6 | MAPK14 | ETSdomainproteinElk-1 | S383 | **2.78** |
| C 7 | CaM-KIV | Histonedeacetylase4 | S467 | **2.77** |
| E14 | CDK2 | B-Myb | T444 | **2.77** |
| L21 | MAP2K1 | HSP22 | S27 | **2.77** |
| O20 | MAPKAPK2 | HSP27 | S15 | **2.77** |
| C 8 | CaM-KIV | E1A-associatedproteinp300 | S24 | **2.77** |
| E 2 | CDK1 | Parathyroidhormonerelatedprotein | T108 | **2.77** |
| K 7 | IKK_group | NuclearfactorNF-kappa-Bp105subunit | S923 | **2.76** |
| O16 | MAPK7 | Serine/threonine-proteinkinaseSgk1 | S78 | **2.76** |
| L 2 | JNK_group | HSF1 | S363 | **2.76** |
| M17 | MAPK_group | Bcl-2 | T69 | **2.75** |
| A 3 | 70-kDakinase | FADD | S194 | **2.75** |
| J 8 | GSK-3_group | HSF1 | S303 | **2.75** |
| M19 | MAPK_group | C/EBPbeta | T235 | **2.75** |
| C24 | CDK | Nestin | T314 | **2.75** |
| D14 | CDK1 | E2F-1 | S332 | **2.75** |
| B23 | CaM-KII_group | Discs,largehomolog1 | S232 | **2.74** |
| I 5 | ERTPK | EGFR | T693 | **2.74** |
| B10 | AuroraBkinase | GFAP | S13 | **2.74** |
| H 5 | CK2 | LupusLaprotein | S366 | **2.74** |
| B12 | AuroraBkinase | Innercentromereprotein | T897 | **2.74** |
| N 1 | MAPK_group | Glucocorticoidreceptor | S226 | **2.73** |
| F12 | CHK1 |  | ST11 | **2.73** |
| J24 | HRI | eIF2alpha | S48 | **2.73** |
| K 1 | IKK_group | IKK-beta | S177 | **2.73** |
| O 4 | MAPK12 |  | ST11 | **2.72** |
| N11 | MAPK_group | Stathmin2 | S62 | **2.72** |
| P 8 | MHCK | MyosinheavychainIB | S315 | **2.72** |
| J20 | GSK-3beta | IPP2 | T72 | **2.71** |
| G 3 | CK1_group | CaseinkinaseI,epsilon | S323 | **2.71** |
| A12 | AMPK_group | HNF4-alpha | S304 | **2.71** |
| D15 | CDK1 | Epsin1 | S357 | **2.71** |
| P17 | NEK6 | Serine/threonine-proteinkinaseSgk1 | S377 | **2.71** |
| O 2 | MAPK12 | Syntrophin | S193 | **2.70** |
| B 9 | AuroraBkinase | Apoptosisinhibitorsurvivin | T117 | **2.70** |
| K23 | IPL1 | DASHcomplexsubunitDAM1 | S20 | **2.69** |
| J17 | GSK-3beta | tau | S574 | **2.69** |
| N18 | MAPK1 | PPARG | S112 | **2.69** |
| C12 | CCDPK | Carboncatabolitederepressingproteinkinase | T210 | **2.69** |
| F 3 | CDK7 | E2F-1 | S403 | **2.68** |
| O14 | MAPK6 | IEX1 | T18 | **2.68** |
| B19 | CaM-KII_group | Adenylatecyclase3 | S1076 | **2.68** |
| N 8 | MAPK_group | PPAR | S12 | **2.68** |
| N 2 | MAPK_group | LIF-R | S1044 | **2.67** |
| B18 | CaM-KI_group | Calcium/calmodulin-dependentproteinkinaseIIgamma | S333 | **2.67** |
| O18 | MAPKAPK2 | Vimentin | S55 | **2.67** |
| A22 | ATM | Rad17 | S646 | **2.66** |
| F14 | CHK2 | BRCA1 | S988 | **2.66** |
| N 9 | MAPK_group | RetinoicacidreceptorRXR-alpha | T82 | **2.66** |
| N 4 | MAPK_group | Mitogen-activatedproteinkinase6 | S189 | **2.66** |
| P20 | NEK9 | Ser/thr-proteinkinaseNek7 | S195 | **2.66** |
| C15 | CDK | Amphiphysin | S272 | **2.66** |
| J 9 | GSK-3_group | Beta-catenin | S33 | **2.65** |
| O19 | MAPKAPK2 | Tyr-3-hydroxylase | S19 | **2.65** |
| F 8 | CDK7 |  | ST11 | **2.63** |
| C23 | CDK | Myogenicfactor3 | S200 | **2.63** |
| E 8 | CDK1 | Sam68 | T317 | **2.62** |
| B 6 | AuroraAkinase | p53 | S215 | **2.62** |
| C20 | CDK | Estrogenreceptoralpha | S104 | **2.62** |
| M20 | MAPK_group | C/EBPepsilon | T74 | **2.62** |
| L22 | MAP2K1 | SMAD2 | T8 | **2.61** |
| E 5 | CDK1 | Ras-relatedproteinRab-5B | S123 | **2.61** |
| M18 | MAPK_group | Beta-ARK-1 | S670 | **2.61** |
| C18 | CDK | DARPP-32 | T75 | **2.61** |
| J 3 | GRK-5 |  | T393 | **2.60** |
| M11 | MAP3K1 | C-ets-2protein | T72 | **2.60** |
| J14 | GSK-3beta | CD227 | S1227 | **2.60** |
| B22 | CaM-KII_group | Dihydropteridinereductase | S223 | **2.59** |
| D 1 | CDK | p53 | S33 | **2.59** |
| D 9 | CDK |  | ST11 | **2.59** |
| C 6 | CaM-KIV | CREB-bindingprotein | S302 | **2.58** |
| I 2 | DNA-PK |  | ST11 | **2.58** |
| C 9 | CaM-KIV |  | ST11 | **2.58** |
| C16 | CDK | BRCA1 | S1497 | **2.57** |
| E 7 | CDK1 | RibonucleotidereductaseM2subunit | S20 | **2.57** |
| A15 | ATM | ATM | S1981 | **2.57** |
| B 4 | AuroraAkinase | BRCA1 | S308 | **2.57** |
| A 5 | AMPK_group | Caspase-6precursor | S257 | **2.57** |
| D 4 | CDK | Ras-relatedproteinRab-4A | S199 | **2.57** |
| F 1 | CDK5 |  | ST11 | **2.57** |
| E 9 | CDK1 | Securin | S165 | **2.56** |
| A18 | ATM | DNAligaseIV | S199 | **2.56** |
| D11 | CDK1 | Caldesmon | T638 | **2.55** |
| B21 | CaM-KII_group | Cofilin2 | S24 | **2.55** |
| C 1 | CaM-KII_group | HSF1 | S230 | **2.55** |
| G24 | CK2 | EGR-1 | S378 | **2.55** |
| D12 | CDK1 | Cutlike1 | S1237 | **2.55** |
| J18 | GSK-3beta | hnRPD | S83 | **2.54** |
| M16 | MAP4K4 | NuclearfactorNF-kappa-Bp100subunit | S865 | **2.54** |
| E10 | CDK1 | Ubiquitin-conjugatingenzymeE2A | S120 | **2.53** |
| M22 | MAPK_group | c-Myb | S532 | **2.53** |
| E13 | CDK2 | B-Myb | T267 | **2.52** |
| O13 | MAPK4 | Mitogen-activatedproteinkinase4 | S196 | **2.52** |
| C21 | CDK | Glutamate[NMDA]receptorsubunitepsilon1 | S1232 | **2.52** |
| C 4 | CaM-KII_group | YL-1protein | T86 | **2.51** |
| I10 | GRK_group | CCR5 | S336 | **2.50** |
| K15 | ILK | Proteinphosphatase1regulatorysubunit14C | T73 | **2.50** |
| B24 | CaM-KII_group | FilaminA | S2522 | **2.49** |
| L11 | MAP2K_group | CDX2 | S60 | **2.49** |
| I 9 | GRK_group | Beta-2adrenergicreceptor | S355 | **2.49** |
| K24 | IPL1 |  | ST11 | **2.48** |
| H21 | DNA-PK | c-Jun | S249 | **2.48** |
| B16 | BCKDK | BCKDHE1-alpha | S333 | **2.46** |
| P 2 | MAPKAPK2 | Tyr-3-hydroxylase | S40 | **2.45** |
| J22 | GSK-3beta | Axin1 | T480 | **2.45** |
| P15 | NEK2 | Retinoblastoma-associatedproteinHEC | S165 | **2.45** |
| E12 | CDK11 | eIF3p47 | S46 | **2.44** |
| M15 | MAP3K8 | MAP3K14 | T559 | **2.44** |
| K20 | IPL1 | DASHcomplexsubunitSPC19 | S107 | **2.43** |
| G 2 | CK1_group | Beta-catenin | S45 | **2.42** |
| L23 | MAP2K1 | UBF-1 | T117 | **2.41** |
| A24 | ATM | Telomericrepeatbindingfactor1 | S219 | **2.41** |
| L 5 | JNK_group |  | ST11 | **2.40** |
| M21 | MAPK_group | C-ets-1protein | T38 | **2.40** |
| P 3 | MAPKAPK2 |  | ST11 | **2.39** |
| E11 | CDK1 |  | ST11 | **2.39** |
| B17 | CaM-KI_group | Acetyl-CoAcarboxylase1 | S25 | **2.39** |
| L20 | MAP2K1 | Caspase9 | T125 | **2.39** |
| D17 | CDK1 | Golgin95 | S25 | **2.38** |
| G 4 | CK1_group | CDK5 | S159 | **2.38** |
| F 7 | CDK7 | CDK4 | T172 | **2.38** |
| L17 | MAP2K_group | Steroidogenicfactor1 | S203 | **2.37** |
| D 5 | CDK | RB | S249 | **2.37** |
| E24 | CDK5 | Nudelikeprotein | S198 | **2.37** |
| F16 | CHK2 | cdc25A | S178 | **2.36** |
| J 6 | GSK-3_group | C/EBPalpha | T222 | **2.36** |
| P 1 | MAPKAPK2 | Vimentin | S38 | **2.35** |
| M23 | MAPK_group | Cytokeratin8 | S431 | **2.35** |
| M 8 | MAP2K4 | MAP2K4 | S257 | **2.35** |
| B15 | AuroraBkinase |  | ST11 | **2.34** |
| A 4 | AFK | Actin,plasmodialisoform | T202 | **2.34** |
| F 2 | CDK7 | CDK7 | S164 | **2.34** |
| E 6 | CDK1 | Replicationfactor-Aprotein2 | S23 | **2.33** |
| G 7 | CK1_group | p53 | T18 | **2.33** |
| M12 | MAP3K7 | SMAD3 | S422 | **2.32** |
| C17 | CDK | CROC4 | S60 | **2.31** |
| F13 | CHK2 | E2F-1 | S364 | **2.31** |
| H24 | DNA-PK | Glucocorticoidreceptor | S508 | **2.30** |
| N 3 | MAPK_group | MAPkinase-activatedproteinkinase2 | T25 | **2.29** |
| L12 | MAP2K_group | Mitogen-activatedproteinkinase3 | T202 | **2.28** |
| J15 | GSK-3beta | glycogensynthase | S641 | **2.27** |
| J13 | GSK-3_group |  | ST11 | **2.26** |
| N10 | MAPK_group | S6K-alpha1 | T359 | **2.25** |
| D 3 | CDK | PP1A | T320 | **2.23** |
| L18 | MAP2K_group | MAP2K1 | S217 | **2.22** |
| G12 | CK2 | HSP90-alpha | S230 | **2.22** |
| C19 | CDK | Disabledhomolog1 | S491 | **2.21** |
| M 1 | MAP2K1 | TranscriptionfactorSpi-B | T56 | **2.21** |
| L14 | MAP2K_group | Mitogen-activatedproteinkinase9 | T404 | **2.20** |
| K 2 | IKK_group | Insulinreceptorsubstrate1 | S268 | **2.19** |
| M10 | MAP2K6 | PAK6 | T564 | **2.18** |
| A17 | ATM | CHK1 | S317 | **2.16** |
| L 6 | KIS | Cyclindependentkinaseinhibitor1B | S10 | **2.16** |
| L 7 | KIS | Synapsin-1 | S438 | **2.15** |
| C 5 | CaM-KII_group |  | ST11 | **2.15** |
| J 1 | GRK-4 | Lipasehormonesensitive | S865 | **2.14** |
| B 5 | AuroraAkinase | Methyl-CpGbindingprotein3 | S24 | **2.14** |
| A16 | ATM | BRCA1 | S1189 | **2.14** |
| I 1 | DNA-PK | SRF | S435 | **2.13** |
| N12 | MAPK_group | TCF-3 | T355 | **2.10** |
| F15 | CHK2 | cdc25c | S216 | **2.09** |
| I12 | GRK_group | MuscarinicacetylcholinereceptorM2 | S232 | **2.08** |
| H22 | DNA-PK | DNA-PKcs | T2609 | **2.08** |
| F 5 | CDK7 | Retinoicacidreceptoralpha | S77 | **2.06** |
| F24 | CK1epsilon | Periodcircadianprotein2 | S662 | **2.05** |
| D 2 | CDK | P70killercellinhibitoryreceptor | S275 | **2.03** |
| L19 | MAP2K_group |  | ST11 | **2.02** |
| M 2 | MAP2K1 | PLCbeta1 | S982 | **2.02** |
| F17 | CHK2 |  | ST11 | **2.02** |
| P13 | MST1 | Ser/thr-proteinkinase4 | T183 | **2.01** |
